# Rapid Fabrication by Digital Light Processing 3D Printing of a SlipChip with Movable Ports for Local Delivery to Ex Vivo Organ Cultures

**DOI:** 10.3390/mi12080993

**Published:** 2021-08-20

**Authors:** Megan A Catterton, Alexander G Ball, Rebecca R Pompano

**Affiliations:** 1 Department of Chemistry, University of Virginia College of Arts and Science, Charlottesville, VA 22904, USA; mac6fa@virginia.edu; 2 Department of Microbiology, Immunology and Cancer Biology, University of Virginia School of Medicine, Charlottesville, VA 22903, USA; agb2kp@virginia.edu; 3 Carter Immunology Center and UVA Cancer Center, University of Virginia, Charlottesville, VA 22903, USA; 4 Department of Biomedical Engineering, University of Virginia School of Engineering and Applied Sciences, Charlottesville, VA 22904-4259, USA

**Keywords:** SLA printing, resin printing, tissue culture, local stimulation, two-phase microfluidics

## Abstract

SlipChips are two-part microfluidic devices that can be reconfigured to change fluidic pathways for a wide range of functions, including tissue stimulation. Currently, fabrication of these devices at the prototype stage requires a skilled microfluidic technician, e.g., for wet etching or alignment steps. In most cases, SlipChip functionality requires an optically clear, smooth, and flat surface that is fluorophilic and hydrophobic. Here, we tested digital light processing (DLP) 3D printing, which is rapid, reproducible, and easily shared, as a solution for fabrication of SlipChips at the prototype stage. As a case study, we sought to fabricate a SlipChip intended for local delivery to live tissue slices through a movable microfluidic port. The device was comprised of two multi-layer components: an enclosed channel with a delivery port and a culture chamber for tissue slices with a permeable support. Once the design was optimized, we demonstrated its function by locally delivering a chemical probe to slices of hydrogel and to living tissue with up to 120 µm spatial resolution. By establishing the design principles for 3D printing of SlipChip devices, this work will enhance the ability to rapidly prototype such devices at mid-scale levels of production.

## 1. Introduction

The ability to produce microchips easily and with minimal manual assembly, while retaining rapid prototyping capabilities, is highly desirable for pushing microfluidic devices past the first hand-built prototype stage [1,2,3]. Scaled-up fabrication is critical to conducting experiments at moderate scale (dozens of devices) and for propagating such technology to collaborators. In particular, this scale of fabrication would be useful for SlipChips, which are two-phase, reconfigurable microfluidic devices [4,5,6,7,8,9]. SlipChips usually comprise two planar components that can be “slipped” relative to one another, contain recessed features to hold droplets or streams of aqueous solution, and are separated by a thin layer of oil [4]. SlipChip devices were first developed in the Ismagilov lab [4] as a new technology to perform in low-resource settings [5,6,7]. The first SlipChips were fabricated from glass plates, which offer ideal surface properties and optical clarity but require wet etching with HF, a hazardous procedure that requires a skilled technician [4,10]. Since then, many different Slip-based designs have evolved, including rotational Slipdisc and paper-based SlipPADs, to perform a wide range of laboratory processes such as PCR, cell culture and local delivery to tissue slices [8,9,11,12,13,14,15,16,17]. Fabrication is especially challenging for novel slip-based devices that have multiple layers per component [9,17]. Although injection molding can simplify fabrication at large scale [18], an alternative method is needed to fabricate SlipChips at a moderate scale, while retaining the ability to rapidly prototype.

Any fabrication system for SlipChips must be able to meet four platform requirements, in addition to producing the specific features needed for the intended application. To prevent the aqueous phase from spreading into the oil-filled gap between components, high capillary pressure at the oil–water interface must be maintained. Therefore, the surfaces in contact with the oil layer must be flat and smooth enough to create a gap height of ~1–10 µm across the entire face of the chip [5]. Furthermore, these surfaces must be hydrophobic; if a fluorinated oil is used [4], then a fluorophilic surface is preferred. Finally, for SlipChips that rely on visual alignment or optical detection, the layers must be optically transparent. 

Considering these requirements, we reasoned that digital light projection (DLP) 3D printing, which uses UV or blue light to cure photocrosslinkable resins layer by layer [19,20], may facilitate SlipChip fabrication and allow for rapid prototyping. This additive method is quickly gaining popularity for fabricating small parts and microfluidic devices, because of both its high feature resolution and reproducibility and its rapid fabrication speed compared to traditional soft-lithography [3,21,22,23]. While 3D printing has not been reported previously for SlipChips, two of the four fabrication requirements are already met. We recently described a method for fluorination of a DLP-printed surface based on solvent-based deposition of a fluoroalkyl silane [24], and others have demonstrated optically transparent parts by printing clear resins on a glass surface to reduce light scattering [25]. 

**Figure 1:**
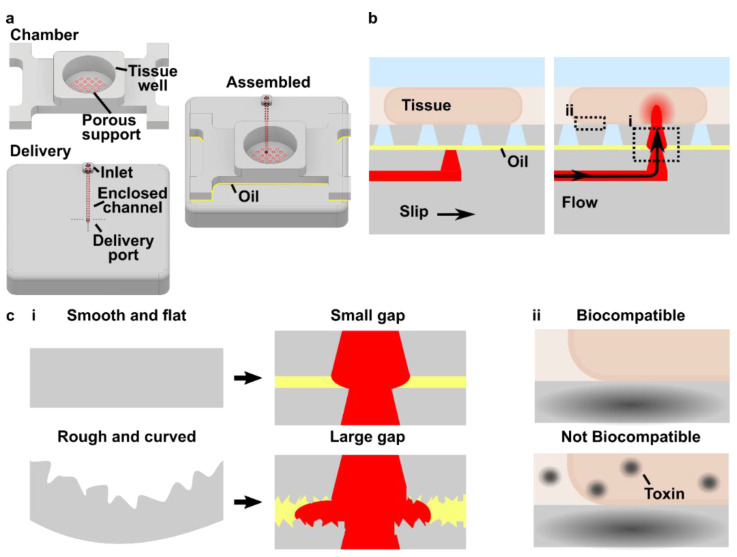
Conceptual design of a 3D printed SlipChip with movable ports. (**a**) AutoCAD Inventor 3D drawing of the chamber and delivery components, with the key features of each component labeled, and a schematic of the device assembled with the oil layer. (**b**) Sideview of the assembled device, loaded with a tissue slice, before (left) and during (right) alignment and fluid delivery into the slice. Boxes show areas highlighted in panel c. (**c**) The two major challenges for developing a movable port SlipChip were to ensure that (**i**) the surfaces were both smooth and flat, thus preventing leaks by minimizing the size of the gap between components, and (**ii**) the material was sufficiently biocompatible.

As a case study for fabrication of a SlipChip by 3D resin printing, we considered a microfluidic movable port device (MP device) previously developed by our lab for local stimulation of ex vivo organ slices at user-selected locations [9]. The MP device is a SlipChip that is comprised of two multilayer components: a bottom component containing a simple enclosed microchannel that terminates in a single, vertical delivery port (delivery component), and a top component featuring a semipermeable tissue culture well (chamber component) (Figure 1a). A bolus of aqueous solution is pumped into a specific region of a tissue slice by aligning the delivery port to a port in the culture well (Figure 1b). Local delivery devices like this one have been used to study intrinsic tissue properties and to screen for potential drugs [9,26,27,28,29,30]. Compared to a device with stationary ports, the SlipChip functionality of the MP device lessens the amount of user handling of a tissue slice and allows more flexible on-demand selection of the delivery region. However, in the original hand-built prototype, an extensive fabrication process limited the accessibility and distribution of the MP device to other labs and collaborators [9]. 

Here, we established an approach to fabricate a 3D printed SlipChip for the first time, using the MP device as a case study. First, we validated the selection of a DLP resin designed for microfluidic devices to meet the optical transparency, surface roughness, surface chemistry, and biocompatibility requirements of the tissue-specific movable port device. Next, the device design was optimized to maximize the functionality of the required ports and channels while minimizing the fabrication time complexity with DLP printing. The ability of the assembled device to deliver aqueous solutions without leaks into the gap was tested, and finally, we tested the ability to stimulate live organ cultures locally and with the position selected on demand. 

## 2. Materials and Methods

### 2.1. Device Design, 3D Printing, and Laser Etching

All 3D printed parts were designed using Autodesk Inventor 2018 (Mill Valley, CA, USA). The CAD files (in Supporting Information) were sliced at 50 µm intervals using MII Utility Shortcut V 3.27 and printed using a CADworks3D M50-405 printer (30 µm xy-resolution, CADworks3D, Toronto, ON, Canada) in BV-007A resin (MiiCraft, via CADworks 3D). The printer setting for the BV-007A resin at a 50 µm slice height was a slow peeling speed, 0.1 mm gap adjustment (unless printing on glass which was 0.27 mm), 1.15 s curing time, 1 base layer, 9.0 s base curing time, 1 buffer layer, and 75% light power. To print parts on glass, a cover glass slide, 36 mm × 60 mm with a thickness of 0.13–0.17 mm (Ted Pella, Redding, CA, USA), was attached to the baseplate by curing a thin layer of BV-007A with a 405 nm UV light (Amazon, Seattle, WA, USA) [25]. The parts were rinsed with methanol (Fisher Chemical, Waltham, MA, USA) and post-cured in a UV light box for 20 s. No additional leaching steps were applied to the printed pieces used in this work. In preliminary experiments, we found that solvent washes at varied temperatures or extended UV light exposure did not substantially improve the biocompatibility of the BV-007A resin. To complete the chamber component, an array of ports with an 80 μm diameter were laser etched (Versa Laser 3.5, Universal Laser Systems, Scottsdale, AZ, USA) into the printed BV-007A part, using a power setting of 7% and a speed of 10%.

### 2.2. Fluorination of Resin Surface and Contact Angle Measurements

Parts printed in BV-007A were silanized using our recently described method [24]. The parts were submerged into a 10% *v*/*v* solution of tridecafluoro-1,1,2,2-tetrahydrooctyl trichlorosilane (Gelest Inc., Morrisville PA, USA) in Fluorinert FC-40 (Sigma Aldrich, St. Louis, MO, USA) for 30 min at room temperature. The surfaces were rinsed with 95% ethanol (Koptec) and DI water and finally dried with a nitrogen gun. 

Surface air–water contact angles and three-phase contact angles were measured on cubic printed pieces (8 × 8 × 15 mm^3^) using a ramé-hart goniometer (model 200-00, ramé-hart instrument co., Succasunna NJ, USA) and DROPimage Advanced software (ramé-hart instrument co., Succasunna, NJ, USA). For consistency, the smooth, flat face of the cube produced against the polytetrafluoroethylene (PTFE) sheet was tested in all cases; this was also the side of the print that faced the oil layer in the SlipChip. The contact angle was measured in triplicate (3 separate printed pieces per condition), by pipetting one 5 µL droplet of 1× phosphate buffered saline (PBS) (Lonza, Walkersville, MD, USA.; DPBS without calcium or magnesium) onto the printed surface. For three-phase contact angle, the printed cube with a droplet was inverted into a cuvette filled with FC-40 oil containing 0.5 mg/mL triethyleneglycol mono[1H,1H-perfluorooctyl]ether (RfOEG). RfOEG was synthesized in house as reported previously (see Supporting Methods) [9,31,32].

### 2.3. Surface Profilometry

To assess surface roughness, the root mean square deviation of the surface height of the printed parts was measured with a Zygo optical surface profilometer (Zygo, Berwyn, PA, USA) at the Nanoscale Materials Characterization Facility at the University of Virginia. Cubes of 8 × 8 × 8 mm^3^ were printed, and surface roughness was measured on all sides, specifically the surfaces printed against the aluminum baseplate or printed against glass, closest to the PTFE sheet at the bottom of the vat, and the sides of the print. As a positive control, a glass microscope slide was also analyzed after plating with 30 nm of Au/Pd by a Technics sputter coater (Technics).

### 2.4. Measurement of Curvature of Printed Pieces

Images of the side profiles of 3D printed 30 × 30 mm^2^ prisms of varied height (2–5 mm) were collected using a Zeiss AxioZoom microscope (Jena, Germany). The displacement from horizontal due to curvature was manually measured in Zen 2 software (Zeiss, Jena, Germany).

### 2.5. Animal Work and Tissue Slice Collection

All animal work was approved by the Institutional Animal Care and Use Committee at the University of Virginia under protocol #4042, and was conducted in compliance with guidelines of the Office of Laboratory Animal Welfare at the National Institutes of Health (United States). Both male and female C57BL/6 mice aged 19–21 weeks (Jackson Laboratory, Bar Harbor, ME, USA) were housed in a vivarium and given water and food ad libitum. Lymph nodes were harvested from the mice following humane isoflurane anesthesia and cervical dislocation. The tissues were sliced according to a previously published protocol [33]. Briefly, peripheral lymph nodes were collected and embedded in 6% *w*/*v* low melting point agarose (Lonza, Walkersville, MD, USA) in 1× PBS. After the agarose had hardened, agarose blocks containing lymph nodes were extracted with a 10 mm tissue punch (World Precision Instruments, Sarasota, FL, USA). The blocks were mounted with super glue on a stage and sliced into 300 μm thick sections using a Leica VT1000S vibratome (Bannockburn, IL, USA) in ice-cold 1× PBS. The lymph nodes were sliced at a speed setting of 90 (0.17 mm/s) and frequency of 3 (30 Hz). Slices were cultured in “complete RPMI”: RPMI 1640 (Lonza, 16-167F) supplemented with 10% FBS (VWR, Seradigm USDA approved, 6 89510-186), 1× L-glutamine (Gibco Life Technologies, 25030-081, Waltham, MA, USA), 50 U/mL Pen/Strep (Gibco), 50 μM beta-mercaptoethanol (Gibco, 21985-023), 1 mM sodium pyruvate (Hyclone, GE USA), 1× non-essential amino acids (Hyclone, SH30598.01), and 20 mM HEPES (VWR, 97064–362). Slices of 6% agarose were collected in a similar manner but were stored in 1×PBS instead of complete media.

### 2.6. Analysis of Tissue Viability

To assess the viability of lymphoid tissue slices after a brief exposure to BV-007A, tissue slices were incubated in 1× PBS in a 3D printed culture well (30 mm × 30 mm × 5 mm printed part, with a central 10 mm-diameter well) for 15 min at room temperature. Then, the slices were moved from the printed substrate into a 24-well plate (VWR) and cultured in “complete media” for 4 h at 37 °C with 5% CO_2_ to allow time for any delayed effects of on-chip exposure, such as toxicity mediated by protein transcription or translation, to occur. Following a previously established protocol [33,34], the viability of live lymph node tissue slices was assessed by flow cytometry. Briefly, individual slices were crushed to generate cell suspensions. Cells were stained with 75 μL of 67 nM Calcein AM (eBioscience, San Diego, CA, USA) in 1× PBS for 20 min at 37 °C. Stained samples were washed by centrifugation at 400 g for 5 min and resuspended in 1× PBS + 2% FBS (flow buffer). 7-AAD (AAT Bioquest, Sunnyvale, CA, USA, 5 μg/mL final concentration) was then added to the cell suspension. The samples were run on a Guava easyCyte 4-color cytometer (EMD Millipore, 6-2L, Burlington, MA, USA) and analyzed using Guava^®^ InCyte™ Software (EMD Millipore, Burlington, MA, USA). Single stain compensation controls were run on cells from crushed lymph node slices. The Calcein-AM single stain contained a 1:1 mixture of Calcein-labelled and unstained live cells. The 7-AAD single stain contained a 1:1 mixture of live and killed cells; the latter were prepared by treating cells with 35% ethanol for 10 min. Calcein positive and 7-AAD negative cells were defined as viable cells.

### 2.7. Assembly and Local Delivery with the 3D Printed Slipchip

Prior to assembling the SlipChip, the channel in the delivery component was filled using pressure-driven flow via a Chemyx syringe pump (Fusion 200, Houston, TX, USA). A 0.5 mg/mL solution of FITC-conjugated dextran (150 kDa and 70 kDa for agarose and tissue deliveries experiments, respectively) was flowed into the channel using a 50 μL Hamilton syringe (model 1705 RN; 26 s gauge, large hub needle) and non-shrinkable PTFE TT-30 tubing (0.012” I.D., 0.009” wall thickness, Weico Wire, Edgewood, NY, USA). Next, 500 µL of FC-40 oil containing 0.5 mg/mL RfOEG was pipetted onto the top face of the filled delivery component. The chamber component was lowered onto the delivery component, and the two components were clamped together with two binder clips, sandwiching a thin layer of oil between them. The culture chamber on the top of the chip was then filled with 1× PBS. A sample of agarose gel or tissue was placed into the chamber and weighed down using a small stainless-steel washer (10 mm O.D. and 5.3 mm I.D., Grainger, Lake Forest, IL, USA). The chamber component was manually slipped relative to the delivery component and visually aligned under a microscope to align to a desired port. To initiate a delivery, the syringe pump was turned on at the desired flow rate. After 5 s, the pump was turned off and the device was slipped away, to reposition for another delivery or to a reach a closed position. After all deliveries were complete, the sample was removed, and the chamber was flushed with 1× PBS and refilled for the next sample. All delivery experiments were performed at room temperature.

All deliveries were monitored in real time using a Zeiss AxioZoom upright microscope with a PlanNeoFluor Z 1×/0.25 FWD 56 mm objective, Axiocam 506 mono camera and HXP 200 C metal halide lamp (Zeiss, Jena, Germany), using filter cubes for GFP (Zeiss filter set #38), and Violet Chroma Filter (49021, ET-EBFP2). Images (16 bit) were collected before, during, and after delivery. During deliveries, time lapse images were collected at 1 s intervals. All images were analyzed in Zen 2 software (Zeiss, Jena, Germany).

### 2.8. Analysis of Delivery Widths

After alignment of the delivery port to an array port, a 5 s pulse of fluorescein (FITC)-labeled 150 kDa dextran was delivered to a 6% agarose slice at flow rates ranging from 0.2 to 1 μL min^−1^ (*n* = 3). After delivery, the device was slipped prior to imaging, to avoid the fluorescent signal from the underlying channel. Delivery width was determined from image analysis as previously described [26]. Briefly, line scans were drawn radially across the delivery region, and the background autofluorescence of the resin was subtracted. The data were fit to a Gaussian curve in GraphPad Prism version 8 (San Diego, CA, USA). The width was defined as two standard deviations of the Gaussian curve.

To fit the curve of the spread of analyte with respect to time, we used a previously published analytical model [9]. First, we assumed that the volume delivered per unit time was described by a cylinder:

(1) $$\left(\frac{1}{4}\right)\pi w^{2}h=Q\Delta t$$

where *w* [μm] is the width (diameter) of the delivery, *h* [μm] is the height of the slice, *Q* [μL/min] is the volumetric flow rate set by the pump, and Δ*t* [sec] is the length of time of delivery. Solving for width gives Equation (2):

(2) $$w=2\sqrt{\frac{Q\Delta t}{\pi h}}$$

### 2.9. Delivery to Lymph Node Tissue

The device was assembled and a lymph node slice was placed into the chamber. A 5 s pulse of FITC-labeled 70 kDa dextran was delivered at a flow rate of 0.25 μL min^−1^. After the first delivery, the device was repositioned and another delivery was performed. This was repeated for four different slices and with slight variations in the number of deliveries on three separate occasions.

## 3. Results

### 3.1. Design Goals for a 3D Printed SlipChip with Movable Ports

The movable port device consisted of two components: a chamber to hold a tissue slice with a porous support in the form of a port array, and a delivery component with an enclosed channel with a small terminating port (Figure 1a). To assemble the device, the microchannel in the delivery component was filled with aqueous solution, and the chamber component was lowered on top while carefully sandwiching a layer of immiscible oil in between. To operate the device, the delivery port was aligned with a port in the array above, and pressure driven flow is used to deliver a short pulse of fluid into the tissue in the chamber (Figure 1b).

Before a MP device could be rapidly fabricated by DLP printing, there were two major challenges to be addressed (Figure 1c). The first challenge, applicable to any 3D printed SlipChip, was to have a small gap height between the printed parts to prevent leaking into the oil layer during delivery. To achieve a small gap height, the two surfaces closest to the oil gap must be both smooth and flat across the width of the component (30 mm) (Figure 1c). Flatness can be challenging because photocurable resins shrink when crosslinked, inducing mechanical stress that warps the print if not addressed in the print design [35]. Furthermore, an array of microscale ports and enclosed microchannel had to be integrated without disrupting the flat surface [36]. The second challenge, specific for biological applications, was biocompatibility of the printed resin with tissue slices housed in the delivery component (Figure 1c). The question of resin toxicity is of great interest to the microfluidics community and is still under active investigation [25,37,38].

### 3.2. Selection of Materials and Print Conditions for Transparency, Smoothness, Fluorination, and Cytocompatibility

**Figure 2:**
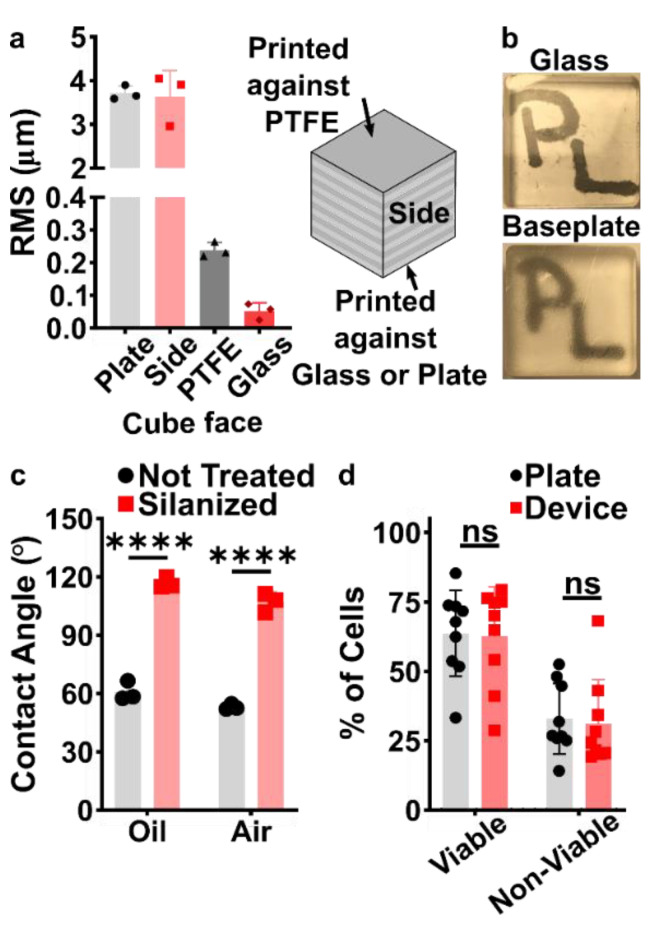
Identification of conditions to ensure suitable surface roughness, optical transparency, surface chemistry, and biocompatibility of the BV-007A printed parts. (**a**) The root mean square (rms) height of surfaces printed against a micro-milled aluminum baseplate, the side of the print, the final print layer closest to the PTFE vat bottom, or printed against a glass slide (*n* = 3, mean ± Std Dev). (**b**) Photos of clear resin parts printed on glass or on the micro-milled aluminum baseplate, showing the optical clarity of the former. (**c**) Three-phase contact angles of a droplet of phosphate buffered saline on a printed part in fluorinated oil or in air, measured before (not treated) and after fluorosilanization of the part. Two-way ANOVA with Tukey’s multiple comparison test, *n* = 3, mean ± Std Dev, **** *p* < 0.0001. (**d**) The percent of viable (Calcein^high^, 7-AAD^low^) and non-viable (7-AAD^high^) cells after 15 min exposure to a 3D printed part or a plate control (*n* = 9, mean ± Std Dev). Two-way ANOVA with Tukey’s multiple comparison test; ns indicates *p* > 0.05.

Before designing the microfluidic device, we first selected and validated a resin for its suitability for the intended use in the SlipChip. We chose to use BV-007A resin because of its ability to generate microfluidic devices with high feature resolution [39,40]. First, we addressed the surface roughness and optical transparency of the DLP printed parts. While the polymeric surface would never be as smooth as glass, prior SlipChips have included microposts to set a defined gap height, e.g., of 2 µm, between two glass components [5]. Therefore, we hypothesized that surface roughness ≤ 2 µm would provide an acceptably small gap height. Surface roughness was expected to differ across the various faces of a printed piece, e.g., the bottom that is printed against the baseplate or against glass, the sides of the print, and the top that prints in contact with the PTFE sheet lining the vat (Figure S1a). As expected, optical profilometry showed that the surface printed against the rough aluminum baseplate and the sides of the printed piece were rough, with RMS (root mean square of surface height) > 3 µm (Figure 2a). The polymeric faces printed against glass and PTFE were much smoother, with RMS ≤ 0.3 µm (Figure 2a). For reference, glass itself had a surface roughness of 5 ± 0.5 nm (*n* = 3). From these data, we concluded that the print for a SlipChip must be oriented such that the surfaces intended to contact the oil gap were printed against glass or the PTFE sheet. Additionally, we also tested for optical transparency, which was desirable for visual alignment of channels and ports in the SlipChip. As previously described [25], printing against glass provided optical transparency, whereas printing against the rough aluminum baseplate yielded an opaque sample (Figure 2b).

To prevent spreading of aqueous solution between the components in the oil gap, the surface chemistry of the chip must be fluorophilic and hydrophobic where it contacts the oil phase. While the BV-007A resin yields parts that are moderately hydrophilic, we recently described a method for fluoroalkyl silanization for SLA resins [24]. Here, this method was applied to silanize the BV-007A, by placing the surface to be silanized in a solution of 10% fluoroalkyl silane in FC-40 oil (see Methods). We confirmed that silanization not only increased the three-phase contact angle of a 1× PBS droplet resting on the surface in air, but also when immersed in FC-40 oil (Figure 2c). The water/oil/resin contact angle of >115° indicated a highly hydrophobic surface [9].

Finally, as the MP device was intended to be used with live organ slices, we sought to identify conditions in which tissue viability was not affected by the BV-007A printed pieces. Ex vivo slices of murine lymph node tissue were used in these experiments, as we have previously characterized local delivery to such tissues [9,26]. During use of the movable port device, the tissue slice is in contact with the surface of the 3D printed chamber for only a few minutes, typically <5 min for alignment and <10 s for the delivery. In separate work, we have shown that multi-hour physical contact of murine splenocytes with parts printed in BV-007A was cytotoxic after just 4 h [41]. Therefore, we restricted this study to an exposure period of 15 min, which represents triple the expected exposure time for tissue spent in contact with the resin during use of the device. Tissue viability after 15 min exposure was comparable to that of off-chip controls (Figure 2d and Figure S2). As this viability test was based on membrane integrity and esterase activity, further tests for cell and tissue function may be appropriate depending on the intended application of the chip. We and others continue to work to identify a resin or a post-print treatment strategy that provides biocompatibility with primary tissues for longer time periods, while still maintaining with the high print resolution of BV-007A [25,37,38,42].

### 3.3. Optimizing the Design and Printability of the Delivery Component

**Figure 3:**
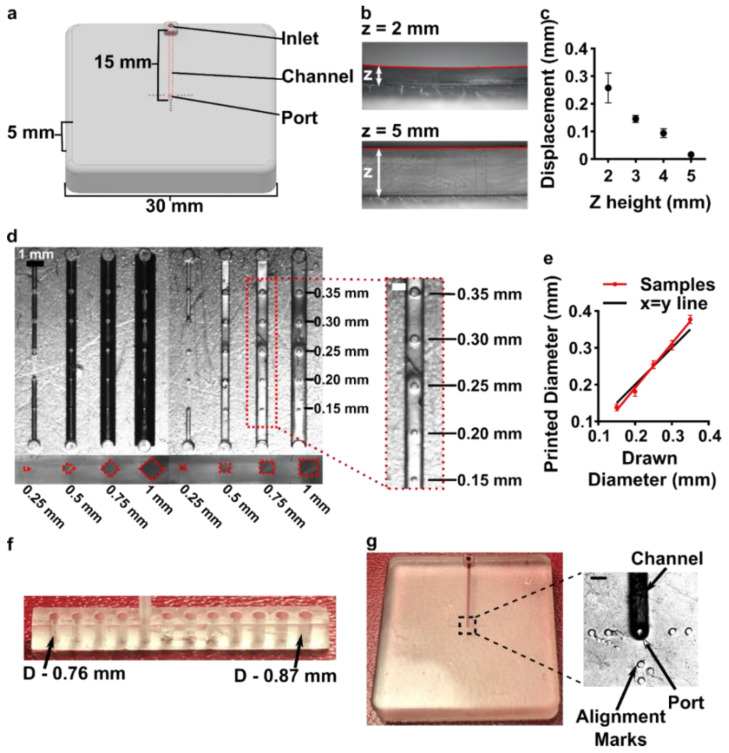
Optimization of the printability of the delivery component of the movable port device. (**a**) AutoCAD inventor drawing of the delivery component. (**b**,**c**) Print warping was minimized by increasing the thickness (z) of the print, analyzed from size-view images (**b**) and quantified in (**c**) as the measured displacement of the center of the print from horizontal (mean ± Std Dev, *n* = 3). (**d**) A top-down image of a printed test piece that was used to determine minimum printable channel and port dimensions. Channel side length varied from 0.25 to 1 mm; cross-sectional shape was diamond or square. The top of each channel included printed ports of 0.15 to 0.35 mm diameter, shown in the inset. (**e**) Printed diameter of port versus drawn dimension (mean ± Std Dev, *n* = 8); linear fit yielded y = 1.2x − 51. The black line shows y = x, for reference. (**f**) A test piece used to optimize the inlet connection to fit a PTFE tubing with the tightest connection. (**g**) Image of the optimized delivery component, with the channel filled with red food dye. Inset shows a micrograph of the delivery port with alignment markers. Scale bar 0.5 mm.

Having identified the material and conditions for SlipChip function, we turned to designing the components of the movable port device. The delivery component required three key features to be printed while maintaining a smooth, flat surface: an interior channel, a delivery port, and an inlet (Figure 3a). Additionally, alignment markers (small inset wells on the top of the component) were included in the design to aid in visual alignment of the device when delivering to opaque tissues. Although it is common practice to print at angle to achieve higher resolution for interior channels (Figure S1b, angled) [43,44], the requirement for smoothness dictated that the design be printed horizontal relative to the baseplate, such that the gap-facing surface was printed against PTFE (Figure S1b, flat). In addition, the requirement for a flat profile to minimize gap height meant contending with shrinkage and associated deformation during photocrosslinking [45]. In addition to rounding the sharp corners, we found that increasing the thickness (z) of the printed part was required to minimize mechanical stress during printing for a part with a 30 × 30 mm^2^ footprint [45,46]. The thickness of the print was varied from 2 to 5 mm, and the delivery component required at least 5 mm thickness to prevent the part from curling (Figure 3b,c).

Having optimized the print geometry and overall dimensions of the piece, the design of the enclosed channels and ports were then optimized to minimize the channel cross-section while retaining printability (Figure 3d). We share these details to aid other researchers who are also working at the limits of the resolution of a 3D printer. To reduce blockage during printing, the channel was positioned close to the top of the part to minimize UV-exposure from subsequent layers, which is a particular issue for transparent resins. Additionally, the length of the channel was minimized (15 mm), because longer channels were more difficult to clear of uncrosslinked resin through the small terminating delivery port. To minimize reagent volume during use, we minimized the cross-sectional area of the channel. In a test piece printed with a series of 15 mm channels of varied cross-sectional size and a square or diamond shape, the minimum cross-section that remained open was 0.5 × 0.5 mm^2^ in both shapes (Figure 3d and Figure S3). Thus, a 0.5 × 0.5 mm^2^ cross-section was selected, and the square was selected over the diamond shape in order to minimize the horizontal width the channel during optical imaging of the device. The diameter of the delivery port was optimized in the same test piece, with a series of ports of varied diameter atop each channel (Figure 3d, inset). All ports with diameter ranging from 0.15 to 0.35 mm were successfully printed, with close fidelity (<10% error) to drawn dimension (Figure 3e). The smallest printable port was 0.138 ± 0.009 mm (drawn diameter 0.15 mm); ports drawn smaller failed to print (data not shown). Finally, we designed a simple press-fit female port to ensure a snug fit with the microfluidic tubing at the inlet (0.78 mm OD PTFE tubing), by printing mock inlets of 0.76–0.87 mm drawn diameter (Figure 3f). A 0.80 mm drawn diameter port was determined to give a snug fit with the tubing. In summary, the optimized design for the inlet, enclosed channel, and terminal delivery port (0.8 mm inlet, 0.5 × 0.5 mm^2^ square channel cross-section, 0.15 mm drawn delivery port) yielded a 3D printed delivery component that could be reproducibly printed and was sufficiently flat and smooth (Figure 3g).

### 3.4. Optimization to Minimize Port Size and Preserve Optical Transparency of the Chamber Component

The top component of the MP device included a chamber (12 mm diameter) to hold tissue samples in media, with a permeable support at the bottom for delivery of fluid from below. Based on our prior work, the ports in the chamber component needed to be in the range of 0.070–0.110 mm, i.e., large enough to minimize flow resistance and small enough to create a localized delivery [9]. The support needed to be transparent for visual alignment, and the requirement for smoothness meant that the bottom of the chamber component needed to be printed against glass or the Teflon vat.

**Figure 4:**
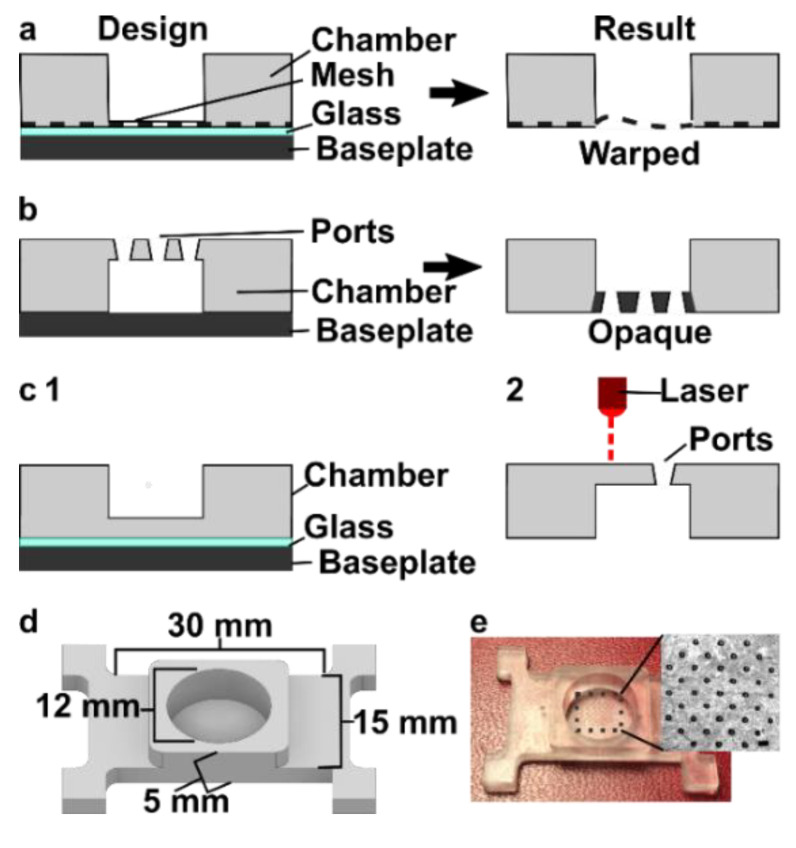
Optimization of the fabrication strategy for a 3D printed chamber with a microporous bottom. Three fabrication strategies were tested: (**a**) incorporating a mesh at the bottom of the device by adhering it to the glass-covered base plate during printing, (**b**) directly printing ports, and (**c**) laser etching the ports after printing. (**d**,**e**) The optimized design of the chamber component shown as an AutoCAD inventor drawing and the 3D printed and laser-etched piece. Inset: Micrograph of the ports etched into the chamber. Scale bar 0.5 mm.

We originally tested a one-step fabrication method for this component, by embedding a membrane or mesh support into the part during 3D printing or by directly printing the port array (Figure 4a,b). We found it simple to embed a nylon or metal mesh in the component by adhering it to the baseplate or glass prior to printing (Figure 4a and Figure S4). Unfortunately, due to resin shrinkage during polymerization, the mesh did not remain taut, preventing its use in the SlipChip. Next, we attempted to directly print the small ports in an array (Figure 3b), but it was challenging to meet the requirements for both small port size and transparency. Orienting the port array against glass on the baseplate proved unfeasible due to the required overexposure of the first layers of the printing, which lowered the spatial resolution in these layers. On the other hand, orienting the port array as an overhang generated ports with an acceptable diameter (~110 µm), but the unsupported overhang led to stretching and distortion, which reduced transparency (Figure 4b).

Since fabricating the port array in a single step proved challenging, we elected to use a two-step process (Figure 4c). First, the chamber component was 3D printed with the solid bottom of the chamber well (200 µm thick) oriented against the glass. Second, a CO_2_ laser was used to etch a port array into the bottom of the chamber. The laser-etched ports had a diameter of 0.081 ± 0.002 mm (*n* = 74), well within the acceptable range, and the entire array was etched in < 1min. Additionally, unlike the accumulation of melted plastic observed when laser etching acrylic [9], there was no deformation of the BV-007A polymer during laser etching on either side of the chamber components (Figure S5), thus minimizing gap height in the SlipChip. The component was sufficiently transparent for visual alignment. Thus, this straightforward fabrication strategy produced a flat, smooth, monolithic part with a well-defined port array, ready for integration into the final SlipChip (Figure 4d,e).

### 3.5. The Assembled 3D Printed SlipChip Delivers Fluid without Leakage into the Gap

**Figure 5:**
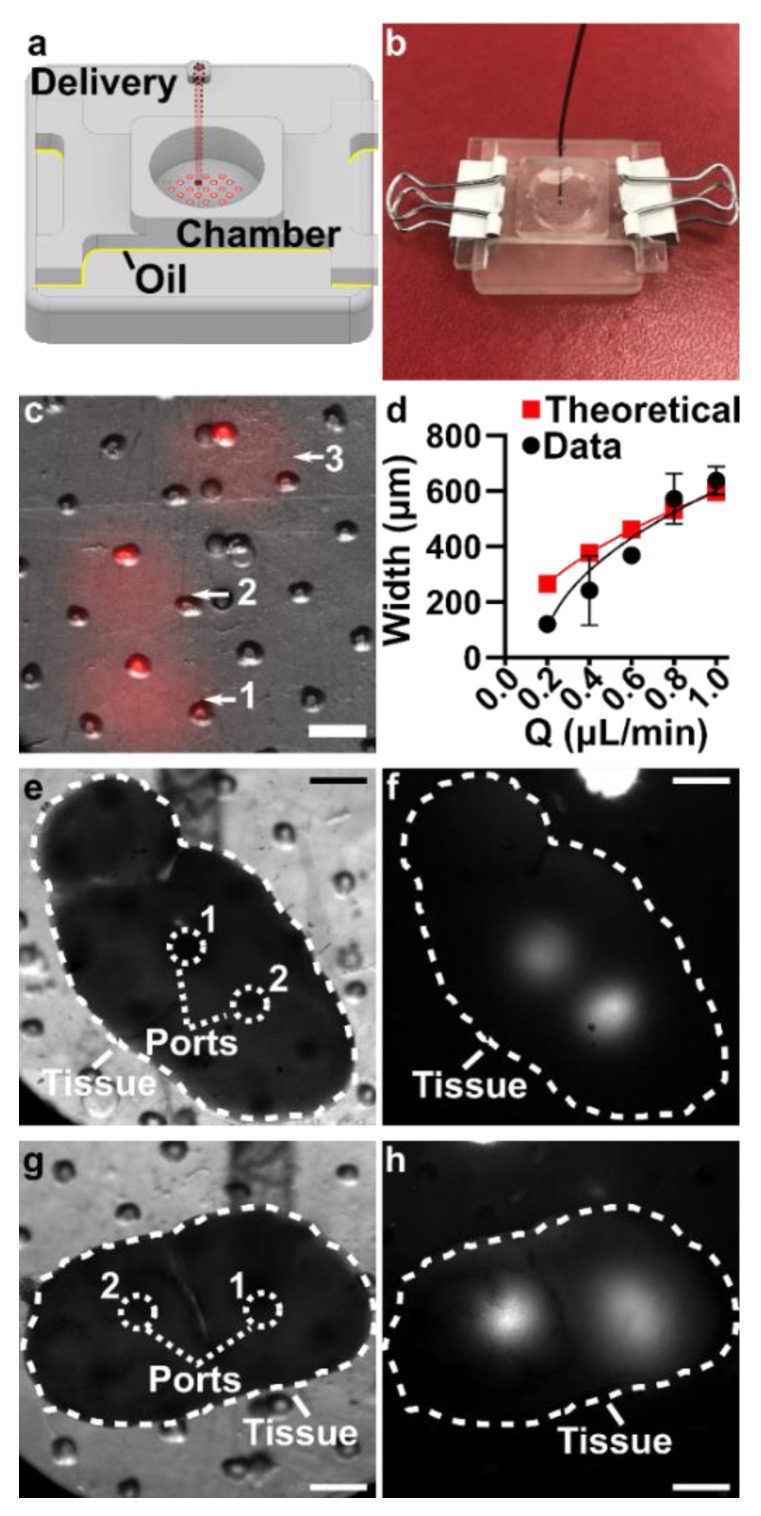
Demonstration of local delivery to slices of hydrogel and live tissue. (**a**) A schematic and (**b**) an image of the assembled two-component device, with red solution filled into the delivery channel for visualization. (**c**) Image after three deliveries of fluorescent dextran (red) to a slab of transparent agarose. Scale bar 500 µm. Numbers indicate the order of the sequential deliveries. Overlaid fluorescent and brightfield data. (**d**) Plot of delivery width as a function of flow rate (mean ± Std Dev, *n* = 3). Experimental data (black circles) were fit with a square root function, $y=663\sqrt{x-0.178}$, R^2^ = 0.85. Red squares indicate theoretical prediction (Equation (2)). (**e**–**h**) Images showing multiple deliveries to replicate tissue slices. (**e**,**g**) Brightfield image of the slice of lymph node tissue (outlined in dashed while line) in the assembled device prior to delivery. The ports used for sequential deliveries (white dotted lines) are indicated, with the order of delivery numbered. Scale bar 500 µm. (**f**,**h**) Fluorescent image after two deliveries of FITC-labeled dextran (white signal) to the live tissue slice.

Having fabricated both components, we assembled the 3D printed SlipChip (Figure 5a,b) and tested its ability to perform local deliveries with leakage of aqueous solution into the oil-filled gap, a critical design goal. To test that the aqueous solution did not leak into the oil gap during use, the delivery port was aligned with a port in the array, and a short pulse of fluorescent dextran solution was delivered to an agarose slice through each of three different array ports (Figure 5c). During and after each delivery, fluorescent and brightfield imaging were used to visually inspect the gap area for the appearance of an interface between aqueous and oil phases, which would indicate a leak. No such interface was observed in 3 separate chip assemblies (9 out of 9 deliveries), indicating a robust capillary-pressure-mediated barrier to leakage. This robust interfacial barrier was also not affected by the size difference between the delivery port (0.138 ± 0.009 mm) and the ports in the chamber array (0.081 ± 0.002 mm).

### 3.6. Validation of Local Delivery to Hydrogel and Tissue Slices on the 3D Printed Chip

Finally, we tested the 3D printed device’s capability to target substructures in gels and tissues via sequential deliveries with high spatial resolution. First, we measured the delivery widths of a model probe, FITC-labeled 150 kDa dextran, delivered in 5 s pulses to 6% agarose, a model 3D culture material, as a function of flow rate (Figure 5d). The delivery width was defined as two standard deviations of the Gaussian fit of a line scan across the delivery [26]. The device was slipped between each pulse to select a new delivery location, taking advantage of the mobility of the SlipChip. As expected, the width of delivery increased with flow rate, and matched well with the predictions from Equation (2), particularly at faster flow rates. At the lowest flow rate, deliveries were achieved as small as 120 µm, which is the highest spatial resolution we have reported, and is small enough to target tissue substructures in the lymph node and other tissues [9,26]. Finally, we tested local delivery to live murine lymph node tissue by using the 3D printed device to deliver multiple times to single tissue slices, again in locations selected on-demand by slipping. Since the tissue is opaque, the alignment of the ports relied on the alignment markers incorporated on the delivery component. Distinct regions within lymph node slices were successfully targeted for local delivery, including, e.g., close together through adjacent ports (Figure 5e,f) or to two different lobes of the lymph node slice (Figure 5g,h).

## 4. Discussion and Conclusions

This paper describes the requirements and fabrication strategy to achieve 3D printed SlipChips for the first time, and demonstrates 3D printing of a SlipChip device with a movable port for local stimulation of organ cultures as a case study. After optimization, DLP 3D printing produced smooth (RMS ≤ 0.3 µm), flat surfaces that were chemically modifiable by fluorination. The resin parts were biocompatible with the short-term (<15 min) exposures needed for local delivery to tissue slices. The delivery component was designed to be printed in a single step and contained an inlet, enclosed channel and small (0.138 ± 0.009 mm) terminating delivery port. The chamber component was produced via 3D printing followed by laser etching, which provided a monolithic culture chamber with an array of 0.081 ± 0.002 mm diameter ports at the bottom, while maintaining a smooth, flat interface for slipping. The device was able to perform multiple slipping and delivery steps without leakage in between the components. The spread of the delivery was dependent on the rate of pump-driven fluid flow, and the resolution was sufficient to target substructures in multiple locations in a tissue slice.

We anticipate that this DLP 3D printing fabrication method will enable the polymeric SlipChips, and in particular the movable port technology, to become accessible to other labs, by greatly simplifying the fabrication steps, materials, and time. Continued advances in biocompatibility of DLP resins [47,48,49] may eventually enable longer-term culture on the 3D printed chip, which was a limitation here. Furthermore, while the current device used binder clips, visual alignment, and manual slipping, 3D printing may enable rapid iteration in the future of other clamping methods and pre-programmed integration with manipulators. Thus, rapid prototyping with DLP 3D printing is expected to accelerate advances in movable port technology as well as other SlipChip device designs.

## Supplementary Materials

The following are available online at https://www.mdpi.com/article/10.3390/mi12080993/s1, Supporting methods: Synthesis of fluorinated surfactant. Supporting figures: Figure S1: Orientation of cubes during printing affects the roughness of the faces of the cube; Figure S2: Gating strategy for analysis of cell viability by flow cytometry; Figure S3: Micrograph showing the cross-sections of internal channels; Figure S4: Mesh incorporation into the chamber component; Figure S5: Port shapes after laser etching of parts 3D printed in BV-007A resin. STL files for each component.
