# Supplementary material for: Rapid Fabrication by Digital Light Processing 3D Printing of a SlipChip with Movable Ports for Local Delivery to Ex Vivo Organ Cultures
Source: Micromachines (Basel). 2021 Aug 20;12(8):993. doi: 10.3390/mi12080993 (PMC8399530; doi:10.3390/mi12080993)
Supplement: Supplementary file 1 [file micromachines-12-00993-s001.zip › micromachines-1322171-supplementary.pdf]

micromachines

Supplementary Materials: Rapid Fabrication by Digital Light Processing 3D Printing of a SlipChip with Movable Ports For Local Delivery to Ex Vivo Organ Cultures

Megan A Catterton [1], Alexander G Ball [2] and Rebecca R Pompano [1,3,4,*]

[1] Department of Chemistry, University of Virginia College of Arts and Science, Charlottesville, VA 22904, USA; mac6fa@virginia.edu
[2] Department of Microbiology, Immunology and Cancer Biology, University of Virginia School of Medicine, Charlottesville, VA, 22903, USA; agb2kp@virginia.edu
[3] Carter Immunology Center and UVA Cancer Center, University of Virginia, Charlottesville, VA 22903, USA
[4] Department of Biomedical Engineering, University of Virginia School of Engineering and Applied Sciences, Charlottesville, VA 22904-4259, VA, USA
* Correspondence: rrp2z@virginia.edu

1. Supporting Methods

1.1. RfOEG synthesis

Triethyleneglycol mono[1H,1H-perfluorooctyl]ether, RfOEG, was synthesized using published protocols [1,2]. RfOEG was stored at a stock concentration of 20 mg/mL in FC-40 (Sigma Aldrich, St. Louis MO, USA) at -20 °C. For experiments, RfOEG was diluted to a working concentration of 0.5 mg/mL in FC-40.

2. Supporting Figures

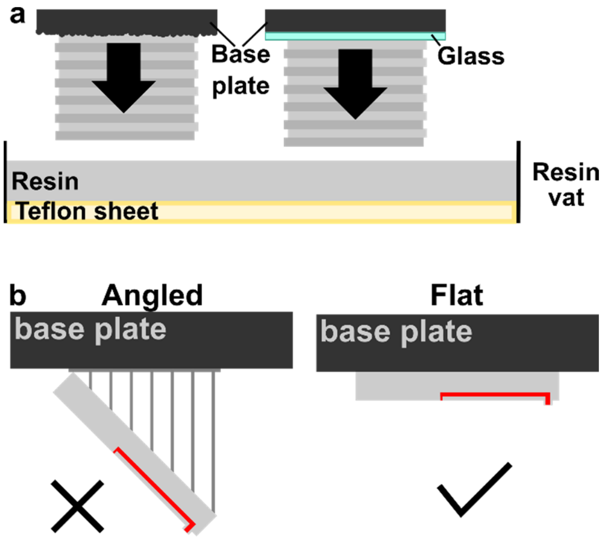

Figure S1. Orientation of cubes during printing affects the roughness of the faces of the cube. (**a**) Cubes printed against an aluminum-based plate (left) or a piece of glass (right). Arrow points towards the top of the cube, which would be the face printed against PTFE sheet. (**b**) Schematic of the printing orientation needed to achieve a smooth surface on the top of the delivery component. The red line represents the enclosed channel. .

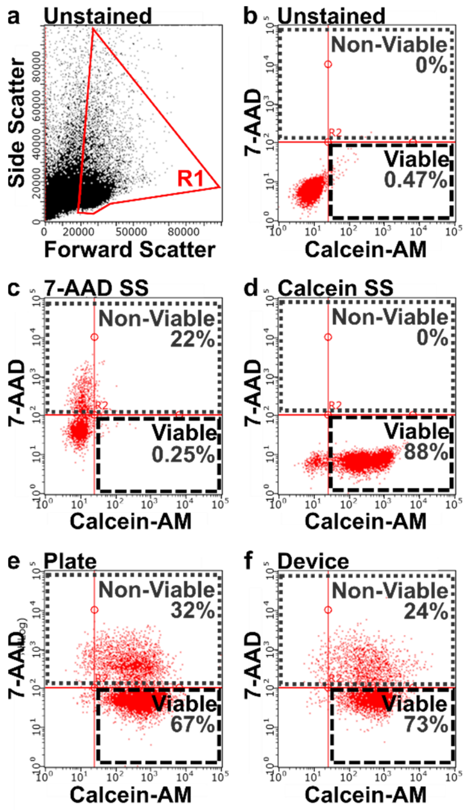

Figure S2. Gating strategy for analysis of cell viability by flow cytometry. (**a**) Cells obtained from crushed tissue slices were gated on scatter and singlets and (**b**–**f**) analyzed for intensity of.

Calcein-AM and 7-AAD. The unstained (b) and single stains (SS) for each dye (c, 7-AAD and d, Calcein-AM) were used to set the position of the quadrants. Any cell high in 7-AAD was considered non-viable and cells high in Calcein-AM and low in 7-AAD fluorescence were considered viable. Examples of a plate cultured control slice (e) and a slice that was exposed to BV-007A (f) are shown.

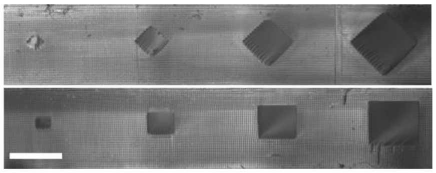

Figure S3. Micrograph showing the internal cross-sections of 3D channels, from the experiment in Figure 3d. Scale bar is 1 mm.

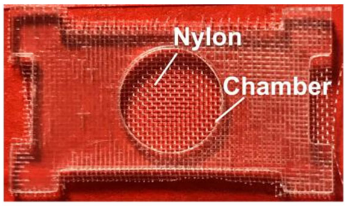

Figure S4. Photo showing mesh incorporated into bottom of the chamber component. For scale, the chamber shown was 12 mm in diameter.

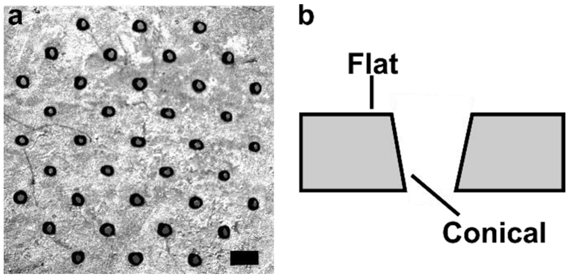

Figure S5. Port shapes after laser etching of parts 3D printed in BV-007A resin. (**a**) An enlarged version of the inset in figure 3 of the laser etch port array. The scale bar is 0.5 mm. (**b**) Schematic of the side view of the created ports. A flat profile was observed above and below the port.

Reference

1. Roach, L.S.; Song, H.; Ismagilov, R.F. Controlling Nonspecific Protein Adsorption in a Plug-Based Microfluidic System by Controlling Interfacial Chemistry Using FluorousPhase Surfactants. *Anal. Chem.* **2005**, *77*, 785–796, doi:10.1021/ac049061w.
2. Cristini, V.; Tan, Y.-C. Theory and Numerical Simulation of Droplet Dynamics in Complex Flows—a Review. *Lab Chip* **2004**, *4*, 257–264, doi:10.1039/B403226H.
